# Loss of MKP-5 promotes myofiber survival by activating STAT3/Bcl-2 signaling during regenerative myogenesis

**DOI:** 10.1186/s13395-017-0137-7

**Published:** 2017-10-18

**Authors:** Kisuk Min, Ahmed Lawan, Anton M. Bennett

**Affiliations:** 1 Department of Pharmacology, Yale University, New Haven, CT 06520 USA; 2 Program in Integrative Cell Signaling and Neurobiology of Metabolism, Yale University, New Haven, CT 06520 USA

**Keywords:** MKP-5, MAP kinase, Regeneration, Myonuclear apoptosis, Mitochondria, STAT3, Bcl-2, Reactive oxygen species

## Abstract

**Background:**

The mitogen-activated protein kinases (MAPKs) have been shown to be involved in regulating myofiber survival. In skeletal muscle, p38 MAPK and JNK are negatively regulated by MAPK phosphatase-5 (MKP-5). During muscle regeneration, MKP-5 is downregulated, thereby promoting p38 MAPK/JNK signaling, and subsequent repair of damaged muscle. Mice lacking MKP-5 expression exhibit enhanced regenerative myogenesis. However, the effect of MKP-5 on myofiber survival during regeneration is unclear.

**Methods:**

To investigate whether MKP-5 is involved in myofiber survival, skeletal muscle injury was induced by cardiotoxin injection, and the effects on apoptosis were assessed by TUNEL assay in wild type and MKP-5-deficient mice. The contribution of MKP-5 to apoptotic signaling and its link to this pathway through mitochondrial function were determined in regenerating skeletal muscle of MKP-5-deficient mice.

**Results:**

We found that loss of MKP-5 in skeletal muscle resulted in improved myofiber survival. In response to skeletal muscle injury, loss of MKP-5 decreased activation of the mitochondrial apoptotic pathway involving the signal transducer and activator of transcription 3 (STAT3) and increased expression of the anti-apoptotic transcription factor Bcl-2. Skeletal muscle of MKP-5-deficient mice also exhibited an improved anti-oxidant capacity as a result of increased expression of catalase further contributing to myofiber survival by attenuating oxidative damage.

**Conclusions:**

Taken together, these findings suggest that MKP-5 coordinates skeletal muscle regeneration by regulating mitochondria-mediated apoptosis. MKP-5 negatively regulates apoptotic signaling, and during regeneration, MKP-5 downregulation contributes to the restoration of myofiber survival. Finally, these results suggest that MKP-5 inhibition may serve as an important therapeutic target for the preservation of skeletal muscle survival in degenerative muscle diseases.

## Background

The mitogen-activated protein kinases (MAPKs) are highly conserved serine/threonine protein kinases that participate in a multitude of signal transduction pathways. MAPKs are activated by a wide variety of extracellular stimuli including mitogens, growth factors, cytokines, and cellular stresses associated with physiological and pathophysiological mechanisms [1–3]. It has been well established that MAPKs play a major role in the control of post-developmental skeletal muscle function [4–8]. The MAPKs are negatively regulated by the MAPK phosphatases (MKPs) through direct dephosphorylation [9]. The MKPs constitute one group of the dual-specificity phosphatases (DUSPs) that exhibit the capacity to dephosphorylate MAPKs on regulatory threonine and tyrosine residues [10–12]. MKP-5 is highly expressed in skeletal muscle suggesting that it plays an important functional role in this tissue [4, 13, 14]. Our group has demonstrated that MKP-5 regulates skeletal muscle function by inactivating both p38 MAPK and c-Jun NH_2_-terminal kinase (JNK) during regenerative myogenesis [4]. MKP-5 negatively regulates muscle stem cells, known as satellite cells (SCs), by inhibiting JNK-mediated expression of cyclin D3 [4]. This inhibition is relieved upon SC activation by the rapid downregulation of MKP-5. As such, genetic loss of MKP-5 in mice results in enhanced SC proliferation and differentiation and improved regenerative myogenesis in response to injury. Furthermore, we identified that MKP-5 is involved in the progression of dystrophic muscle disease [4]. However, a complete understanding of how MKP-5 regulates myofiber integrity remains to be defined.

The restoration of damaged skeletal muscle function requires the balance between the regenerative capacity and the rate of apoptosis [15–17]. Mitochondria play a pivotal role in both regeneration and apoptosis in skeletal muscle following injury [18–20]. While mitochondrial biogenesis is required for skeletal muscle regeneration [21–23], aberrant mitochondria-mediated apoptosis has been suggested to be responsible for certain skeletal muscle diseases [24–26]. Apoptosis is required to regulate development and tissue homeostasis in multicellular organisms. However, deregulated apoptotic cell death can promote a variety of pathologies [27–29].

Growing evidence shows that interleukin-6 (IL-6) and the signal transducer and activator of transcription 3 (STAT3) are required for not only SC activation but also prevention from mitochondria-mediated apoptosis in several tissues [30–32]. In this regard, our group recently demonstrated that MKP-5 regulates myogenesis by mediating MAPK-dependent phosphorylation of the guanine-nucleotide exchange factor for Rab3A (GRAB), which is responsible for the secretion of IL-6. MKP-5-deficient mice, as a result, express increased circulating levels of IL-6 and increased STAT3 activation [33]. Activated STAT3 couples to the apoptotic pathway by upregulating the anti-apoptotic transcription factor, Bcl-2 [34, 35]. Bcl-2 is localized to the outer membrane of mitochondria which inhibits activation of pro-apoptotic proteins, release of cytochrome c and reactive oxygen species (ROS) from permeabilized mitochondrial membrane, thereby preventing mitochondria-mediated apoptosis [36, 37]. Based upon these collective observations, the aim of this study was to investigate whether MKP-5 regulates myofiber survival and to further discern whether it does so through pathways involving mitochondria-mediated programmed cell death.

## Methods

### Animal experiments

MKP-5 knockout mice were generated as described previously [38]. Yale University Institutional Animal Care and Use Committee approved all procedures. Skeletal muscle damage was induced by intramuscular injection of 300 μL cardiotoxin (Sigma-Aldrich, 0.1 mg/mL in PBS) into the gastrocnemius/soleus muscles, after anesthesia by administration of 10 mg/kg ketamine and 1 mg/kg xylazine. Soleus muscle at 10 days after injury was removed and rapidly frozen in liquid nitrogen and stored at − 80°C for subsequent biochemical analyses.

### Reagents and antibodies

All reagents were purchased from standard chemical vendors. The following antibodies were used. Phospho-JAK1 (3331), JAK1 (3344), JAK2 (3230), phospho-STAT3 (Y705) (9145), phospho-STAT3 (S727) (9134), STAT3 (4904), phospho-p38 MAPK (9215), phospho-JNK1/2 (4668), cleaved caspase-3 (9664), cleaved caspase-8 (9429), cleaved caspase-9 (9509), and Erk (9107) were obtained from Cell Signaling Technology. p38 MAPK (sc-535), JNK (sc-571), phospho-JAK2 (sc16566), and Bcl-2 (sc-492) were obtained from Santa Cruz Biotechnology. 4-HNE (ab46545) was obtained from Abcam and catalase (C0979) from Sigma-Aldrich.

### Mitochondrial respiration

Soleus muscle was dissected and placed on a plastic Petri dish containing ice-cold buffer X (60 mM K-MES, 35 mM KCl, 7.23 mM K_2_EGTA, 2.77 mM CaK_2_EGTA, 20 mM imidazole, 0.5 mM DTT, 20 mM taurine, 5.7 mM ATP, 15 mM PCr, and 6.56 mM MgCl_2_, pH 7.1). The muscle was then cut down to fiber bundles (2–3 mg wet wt). The muscle fiber bundles were gently separated in ice-cold buffer X to maximize surface area of the fiber bundle. To permeabilize the myofibers, each fiber bundle was incubated in ice-cold buffer X containing 50 μg/mL saponin on a rotator for 30 min at 4 °C. The permeabilized muscle bundles were then washed in ice-cold buffer Z (110 mM K-MES, 35 mM KCl, 1 mM EGTA, 5 mM K_2_HPO4, and 3 mM MgCl_2_, 0.005 mM glutamate, and 0.02 mM malate and 0.5 mg/mL BSA, pH 7.1).

Mitochondrial respiration was measured at 37 °C in buffer Z using the Oroboros O2K oxygraph. After the respiration chamber was calibrated, permeabilized fiber bundles were incubated with 2 mL of respiration buffer Z containing 20 mM creatine to saturate creatine kinase activity. Flux through complex I was measured using 5 mM pyruvate and 2 mM malate. ADP-stimulated respiration (state 3) was measured by the addition of 500 μM ADP to the respiration chamber. Basal respiration (state 4) was determined in the presence of 10 μg/mL oligomycin to inhibit ATP synthesis. The respiratory control ratio (RCR) was calculated by dividing state 3 by state 4 respiration.

### Mitochondrial hydrogen peroxide (H_2_O_2_) generation

Isolated mitochondrial H_2_O_2_ release was measured using Amplex Red (Molecular Probes, Eugene, OR) as described previously [39]. Mitochondrial ROS measurements are based on the concept that HRP catalyzes the H_2_O_2_-dependent oxidation of non-fluorescent Amplex Red to fluorescent resorufin red. Superoxide dismutase was added to the preparation to convert all superoxide into H_2_O_2_. Fluorescence was measured with excitation at 544 nm and emission at 590 nm immediately after addition of mitochondria. Fluorescence values were converted to H_2_O_2_ release with a standard curve. Rates of H_2_O_2_ production were normalized to mitochondrial protein.

### Terminal deoxynucleotidyl transferase dUTP nick end labeling

Myonuclear apoptosis was determined by terminal deoxynucleotidyl transferase dUTP nick end labeling (TUNEL) using a histochemical fluorescent detection kit (Roche Applied Scientific; Indianapolis, IN). Briefly, 10 μm tissue sections were fixed using a 4% formaldehyde solution, washed and permeabilized with 0.1% Triton X-100 in 0.1% sodium citrate solution. Tissue sections were stained with a dystrophin antibody (Thermo Fisher Scientific) to visualize the sarcolemma membrane. Following the dystrophin staining, tissue sections were then incubated with the TUNEL enzyme label solution and sealed with a Vectashield 4′,6-diamidino-2-phenylindole (DAPI) mounting medium for detection of nuclei (Vector Laboratories; Burlingame, CA). TUNEL-stained tissue sections were imaged using a Zeiss Axiovert S100 fluorescent microscope using Axiovision software (Zeiss). The numbers of TUNEL and DAPI-positive nuclei either within or on the dystrophin-labeled membrane were counted in 10 fields for each sample.

### Evans blue dye uptake

The loss of skeletal muscle fiber membrane integrity was measured by Evans blue dye uptake. After 10 days of cardiotoxin injection, 1% Evans blue dye dissolved in sterile saline was administered by intraperitoneal injection at a dose of 1% of body weight. Twenty-four hours later, muscles were dissected and snap frozen in isopentene precooled in liquid nitrogen. Muscle cryosections of 10 μm thickness were cut, and Evans blue dye uptake was evaluated using Axiovision software (Zeiss).

### RNA extraction and real-time PCR analysis

RNA was isolated from soleus muscle of *mkp-5* ^*+/+*^ and *mkp5* ^*−/−*^ mice using a RNeasy kit (Qiagen, CA) according to the manufacturer’s instructions. A total of 1 μg RNA was reverse transcribed to generate cDNA using a reverse transcriptase PCR kit (Applied Biosystems, CA). Real-time quantitative PCR was carried out using the Applied Biosystems 7500 Fast real-time PCR system, using TaqMan and SYBR green gene expression master mix. TaqMan primers and gene expression master mix from Applied Biosystems were used for Tfam (Mn00447485_m1), NRF-1 (Mn00447996_m1), Mfn2 (Mn01255785), GABPα (Mn00484598_m1), Ndufs1 (Mn00523631_m1), Ndufs5 (Mn00452592_m1), and PGC1-α (Mn01208835_m1) mRNA quantitation. Primers and SYBR green PCR master mix (Applied Biosystems) were used for PRC (5′- TGGACGCCTCCCTTATATCCC and 3′- TGTGAGCAGCGACATTTCATTC). All relative gene expression levels were analyzed using the ΔC_*T*_ method and normalized to 18S rRNA expression.

### Mitochondrial DNA quantification

Total DNA was isolated from soleus muscle from *mkp-5* ^*+/+*^ and *mkp5* ^*−/−*^ mice using QIAamp DNA mini kit (Qiagen) according to the manufacturer’s instructions. Mitochondrial DNA (mtDNA) was quantified by qRT-PCR using primers amplifying the D-loop region on mtDNA (forward primer: 5′- AATCTACCATCCTCCGTGAAACC-3′, reverse primer: 5′- TCAGTTTAGCTACCCCCAAGTTTAA-3′) relative to the nuclear Tert (forward primer: 5′- CTAGCTCATGTGTCAAGACCCTCTT-3′, reverse primer: 5′- GCCAGCACGTTTCTCTCGTT-3′).

### Biochemical analysis

For immunoblotting, soleus muscles were homogenized and lysed on ice in lysis buffer containing 100 mM Tris HCl (pH 7.4) and 25 mM EDTA. C2C12 myoblasts were lysed on ice in lysis buffer containing 50 mM Tris-HCl (pH 7.8), 150 mM NaCl, 1 mM EDTA, 1% Triton X-100, 0.5% sodium deoxycholate, and 0.1% SDS. Lysis buffers were supplemented with protease and phosphatase inhibitors (1 mM Na_3_VO_4_, 10 mM NaF, 1 mM benzamidine, 1 mM phenylmethylsulfonyl fluoride, 1 μg/mL pepstain A, 5 μg/mL aprotinin, 5 μg/mL leupeptin). Tissue or cell lysates were incubated at 4 °C for 30 min and clarified by centrifugation at 14,000 rpm at 4 °C for 10 min. The protein concentration was determined using the bicinchoninic acid (BCA) reagent according to the manufacturer’s instructions (Pierce). Lysates were resolved by SDS-PAGE and transferred onto Nitrocellulose membranes (Bio-Rad). Membranes were blocked with 5% non-fat dry milk or 5% BSA in Tris-buffered saline/Tween-20 (TBST) for 1 h at room temperature. Primary antibodies were diluted in 5% non-fat dry milk or 5% BSA in TBST. After primary antibody incubations, overnight at 4 °C, membranes were washed in TBST three times for 10 min. The membranes were then incubated in secondary antibodies (Cell Signaling Technology) followed by enhanced chemiluminescence detection.

### Cell culture and transient transfections

C2C12 myoblasts were cultured in DMEM supplemented with 10% fetal bovine serum, 1% penicillin-streptomycin, and 1% sodium pyruvate at 37 °C and transfected with constitutively active mutants of MKK6 (EE) and MKK7 (DD) in pcDNA3 obtained from Addgene using Lipofectamine 2000. After 24 h transfection, myoblasts were shifted to differentiation medium (DMEM containing 2% horse serum, 1% penicilin-streptomycin, and 1% sodium pyruvate). Differentiated cells were lysed 48 h later using lysis buffer containing 50 mM Tris-HCl (pH 7.8), 150 mM NaCl, 1 mM EDTA, 1% Triton X-100, 0.5% sodium deoxycholate, and 0.1% SDS.

### Statistical analysis

All data represent the means ± standard errors of the means (SEM). Differences between groups were assessed by a Student’s *t* test or analysis of variance (ANOVA) with Tukey’s multiple comparisons test using Prism software (GraphPad Software).

## Results

### Reduced myofiber damage and myonuclear apoptosis in regenerating skeletal muscle of MKP-5-deficient mice

**Fig. 1:**
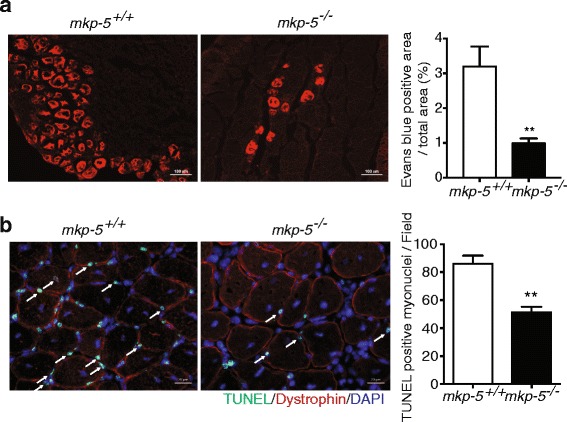
Protection from myofiber damage and myonuclear apoptosis in MKP-5-deficient mice in response to injury. Representative image of sections from the soleus muscle of *mkp-5* ^*+/+*^ and *mkp-5* ^*−/−*^ mice at 10 days after cardiotoxin (CTX)-induced injury (**a**) Representative image of Evans blue dye uptake in the soleus muscle of *mkp-5* ^*+/+*^ and *mkp-5* ^*−/−*^ mice at 10 days after CTX-induced injury. The graph (right panel) represents quantitation showing the percentage of Evans blue dye area in the soleus section in each genotype. Scale bar: 100 μm. (**b**) Stained with terminal deoxynucleotidyl transferase dUTP nick end labeling (TUNEL) to detect for apoptotic nuclei (arrows). Myofibers were stained with dystrophin (red) and nuclei stained with DAPI (blue). Nuclei either within or on the dystrophin boundaries of the myofiber were defined as myonuclei and quantitated. The graph (right panel) represents quantitation showing the TUNEL-positive myonuclei in each genotype. Scale bar: 20μm

The restoration of damaged skeletal muscle depends upon the balance between the regenerative capacity of skeletal muscle and the rate of skeletal muscle death [15–17]. We have demonstrated that MKP-5 negatively regulates regenerative myogenesis in skeletal muscle [4]. MKP-5-deficient mice exhibit increased regenerative myogenesis, in part, due to enhanced SC proliferation and differentiation [4]. However, the effects of MKP-5 deficiency on myofiber survival have yet to be determined. Therefore, we investigated the extent to which MKP-5 is involved in skeletal muscle survival during regenerative myogenesis in MKP-5-deficient mice. Regenerative myogenesis was induced by cardiotoxin-mediated injury in *mkp-5* ^*+/+*^ and *mkp-5* ^*−/−*^ mice (Fig. 1). First, we used Evans blue dye uptake to assess skeletal muscle membrane integrity in uninjured and regenerating skeletal muscle from both *mkp-5* ^*+/+*^ and *mkp-5* ^*−/−*^ mice. In uninjured soleus muscles of both *mkp-5* ^*+/+*^ and *mkp-5* ^*−/−*^ mice as expected, Evans blue dye uptake was undetectable. However, we found that Evans blue dye uptake was significantly reduced in skeletal muscle from *mkp-5* ^*−/−*^ mice as compared with *mkp-5* ^*+/+*^ mice at 10 days after injury (Fig. 1a). These results demonstrate that loss of MKP-5 protects against myofiber damage in response to injury. In order to further examine the nature of the reduced myofiber damage, we analyzed whether myofiber damage occurred as a result of reduced myofiber death. To assess this, we employed TUNEL staining as a measure of apoptotic cell death in myonuclei. In uninjured skeletal muscle, the frequency of TUNEL-positive myonuclei in skeletal muscle sections was undetectable in both *mkp-5* ^*+/+*^ and *mkp-5* ^*−/−*^ mice. We found that skeletal muscle derived from *mkp-5* ^*−/−*^ mice undergoing regeneration exhibited significantly reduced levels of TUNEL-positive myonuclei (Fig. 1b). These findings demonstrate that skeletal muscle in MKP-5-deficient mice exhibit reduced myonuclear apoptosis during regeneration.

**Fig. 2:**
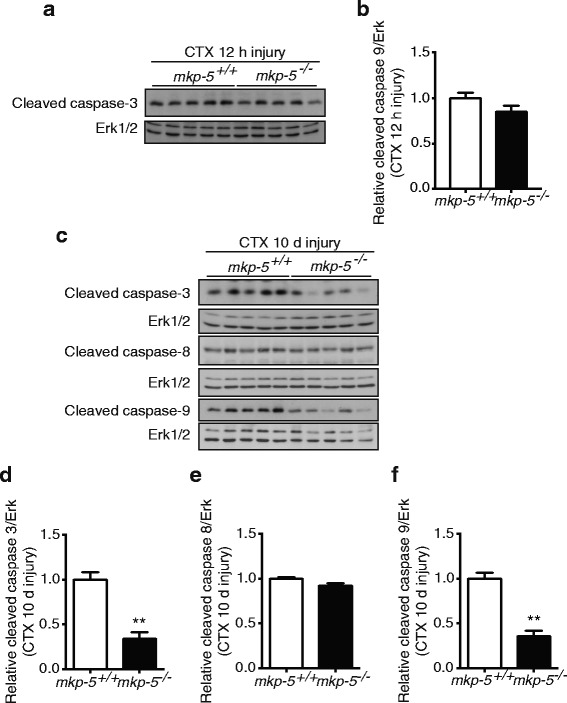
Impaired activation of caspases in skeletal muscle of MKP-5-deficient mice in response to injury. (**a**, **b**) Muscle lysates prepared from *mkp-5* ^*+/+*^ and *mkp-5* ^*−/−*^ mice were immunoblotted for the expression of the cleaved (active) forms of caspase-3 in soleus muscle at 12 h after CTX-induced injury. The expression of the cleaved (active) forms of caspase-3, caspase-8 and caspase-9 was measured in soleus muscle at 10 days after CTX-induced injury (**c**, **d**, **e**, **f**). Graphs represent quantitation of the cleaved form of the indicated caspases normalized to Erk1/2. Values are means ± standard errors of the means (SEM). **P < 0.01, *n* = 5 per genotype

Apoptosis is regulated by highly coordinated processes that involves the activation of cysteine proteases called caspases [27]. Caspase-3 activation is responsible for DNA fragmentation and myonuclear apoptosis [27, 40]. We measured the cleaved, active form, of caspase-3 in skeletal muscle from *mkp-5* ^*+/+*^ and *mkp-5* ^*−/−*^ mice following CTX injection. Since myofiber apoptosis occurs at an early time point in response to injury, we measured cleaved caspase-3 in skeletal muscle at 12 h after CTX injection. Our results revealed that activation of caspse-3 in skeletal muscle derived from *mkp-5* ^*+/+*^ and *mkp-5* ^*−/−*^ mice was not different at 12 h after injury (Fig. 2a, b). However, activation of caspse-3 was significantly attenuated in skeletal muscle from *mkp-5* ^*−/−*^ mice as compared with *mkp-5* ^*+/+*^ mice at 10 days after injury (Fig. 2c, d). To determine whether MKP-5 induces apoptosis through the extrinsic and/or intrinsic pathway, we measured activation of caspase-8 and caspase-9 in regenerating skeletal muscle from *mkp-5* ^*+/+*^ and *mkp-5* ^*−/−*^ mice. These data show that activation of caspase-9 was significantly inhibited in regenerative skeletal muscle from *mkp-5* ^*−/−*^ mice as compared with *mkp-5* ^*+/+*^ mice, whereas activation of caspase-8 in response to injury was unaffected (Fig. 2c, e, and f). These findings demonstrate that MKP-5 deficiency reduces apoptosis during skeletal muscle regeneration through the intrinsic apoptotic pathway.

### Improved mitochondrial function in skeletal muscle of MKP-5-deficient mice

**Fig. 3:**
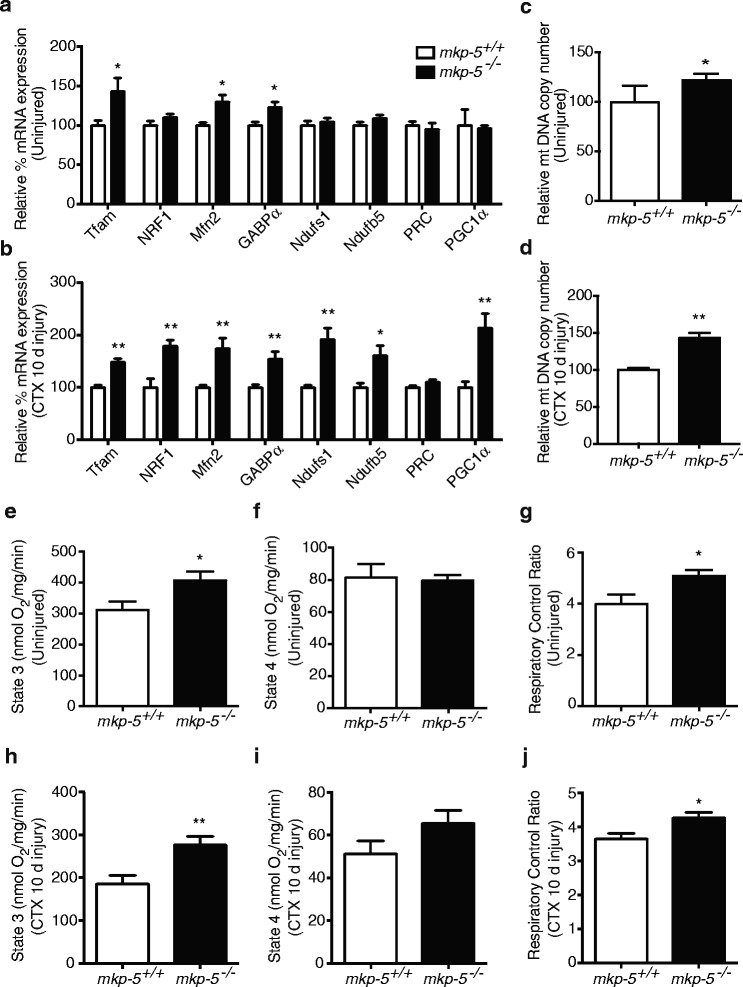
Increased mitochondrial biogenesis and respiratory function in skeletal muscle of MKP-5-deficient mice. Relative mRNA expression of genes associated with mitochondrial biogenesis in soleus muscle in (**a**) uninjured and (**b**) 10 days after CTX-induced injury. Mitochondrial DNA content determined by mitochondrial DNA copy number in the soleus muscle of (**c**) uninjured and (**d**) 10 days after CTX-induced injury. ADP-stimulated respiration (state 3), basal respiration (state 4), and respiratory control ratio (RCR; state 3/state 4) in soleus muscle in (**e**, **f**, **g**) uninjured and at (**h**, **i**, **j **) 10 days after CTX-induced injury. *P < 0.05, **P < 0.01, *n* = 5 per genotype

Our results suggest that MKP-5 is required for the regulation of the intrinsic apoptotic pathway in regenerating skeletal muscle. Given that mitochondria contribute to the activation of anti-apoptotic and pro-apoptotic proteins and are the organelle from which the activation of caspases are induced [18, 27, 41], we investigated whether MKP-5 affects mitochondrial homeostasis during skeletal muscle regeneration. First, we measured the expression of genes associated with mitochondrial biogenesis in regenerating skeletal muscle from *mkp-5* ^*+/+*^ and *mkp-5* ^*−/−*^ mice by quantitative PCR. We found that in uninjured skeletal muscle from *mkp-5* ^*−/−*^ mice, genes such as the mitochondrial transcription factor A (Tfam), mitofusin-2 (Mfn2), and GA-binding protein transcription factor alpha (GABPα) were upregulated as compared with *mkp-5* ^*+/+*^ mice (Fig. 3a). In response to injury, genes associated with mitochondrial biogenesis were more profoundly elevated in skeletal muscle from *mkp-5* ^*−/−*^ mice compared with *mkp-5* ^*+/+*^ mice (Fig. 3b). We determined the levels of mitochondrial DNA (mtDNA), as a marker of mitochondrial content, in skeletal muscle from *mkp-5* ^*+/+*^ and *mkp-5* ^*−/−*^ mice. These results showed that mitochondrial DNA content was significantly increased in both uninjured and regenerating skeletal muscle from *mkp-5* ^*−/−*^ mice as compared with *mkp-5* ^*+/+*^ mice (Fig. 3c, d). Therefore, during skeletal muscle regeneration, MKP-5-deficient mice exhibit increased mitochondrial biogenesis.

To further substantiate the interpretation that MKP-5 deficiency results in increased mitochondrial function, we measured mitochondrial respiration in permeabilized myofibers from *mkp-5* ^*+/+*^ and *mkp-5* ^*−/−*^ mice. Our results showed that the ADP-stimulated respiration (state 3) was significantly increased in permeabilized myofibers from uninjured and injured *mkp-5* ^*−/−*^ mice as compared with *mkp-5* ^*+/+*^ mice (Fig. 3e, h). Basal respiration (state 4) was unaltered between *mkp-5* ^*+/+*^ and *mkp-5* ^*−/−*^ mice in uninjured and injured skeletal muscle (Fig. 3f, i). The respiratory control ratio (RCR), which is an indicator of mitochondrial function, was significantly increased in myofibers from *mkp-5* ^*−/−*^ mice as compared with *mkp-5* ^*+/+*^ mice (Fig. 3g, j). Collectively, these findings suggest that improved mitochondrial function in skeletal muscle of MKP-5-deficient mice may contribute to the reduced levels of apoptosis during regenerative myogenesis.

### Reduced oxidative stress in regenerating skeletal muscle of MKP-5-deficient mice

**Fig. 4:**
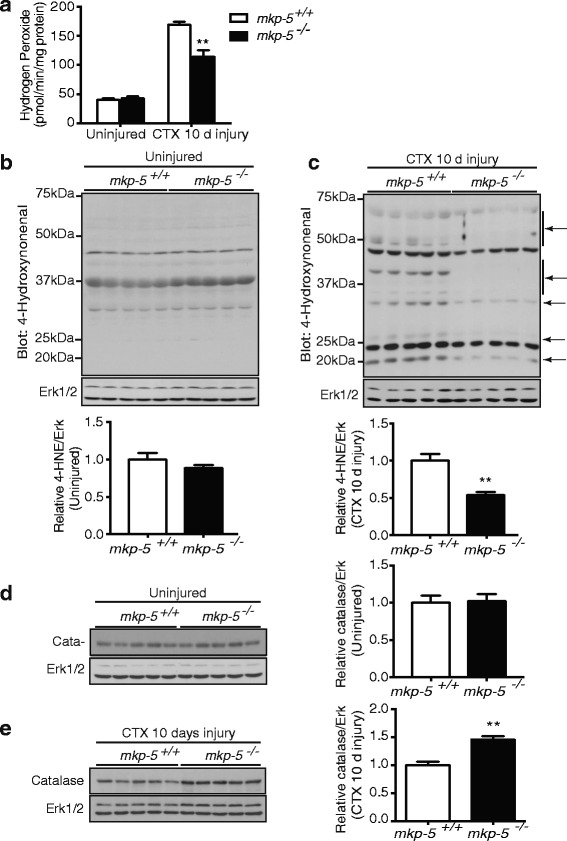
MKP-5 deficiency in regenerating skeletal muscle reduces oxidative damage. (**a**) Hydrogen peroxide release from isolated mitochondria derived from soleus muscle of uninjured and 10 days after CTX-induced injury of *mkp-5* ^*+/+*^ and *mkp-5* ^*−/−*^ mice. 4-HNE-conjugated protein content in soleus muscle was determined by immunoblotting using anti-4-HNE antibodies in (**b**) uninjured skeletal muscle and (**c**) 10 days after CTX-induced injury of skeletal muscle. The graphs below represent the relative intensity of 4-HNE-conjugated proteins quantitated by densitometric analysis of equal lane areas normalized to Erk1/2 levels. Catalase expression in soleus muscle was determined using anti-catalase antibodies in (**d**) uninjured and (**e**) 10 days after CTX-induced injury. The graphs represent quantitation of catalase expression normalized to Erk1/2. Values are means ± standard errors of the means (SEM). **P < 0.01, *n* = 5 per genotype

Mitochondria are one of the major sources of reactive oxygen species (ROS) in skeletal muscle under a variety of pathologies [18, 40, 42]. Increased mitochondrial ROS emission is an important contributor to skeletal muscle apoptosis [18, 43, 44]. Therefore, in order to determine whether MKP-5 deficiency contributes to reduced apoptosis via mechanisms involving mitochondrial ROS production during regenerative myogenesis, we assessed hydrogen peroxide (H_2_O_2_) release from mitochondria isolated from skeletal muscle of *mkp-5* ^*+/+*^ and *mkp-5* ^*−/−*^ mice. In uninjured skeletal muscle of *mkp-5* ^*+/+*^ and *mkp-5* ^*−/−*^ mice, we found that the levels of mitochondria-generated H_2_O_2_ were equivalent between the groups (Fig. 4a). Although mitochondria-generated H_2_O_2_ was dramatically increased in injured skeletal muscle from both *mkp-5* ^*+/+*^ and *mkp-5* ^*−/−*^ mice, mitochondria-generated H_2_O_2_ in skeletal muscle from *mkp-5* ^*−/−*^ mice was significantly less (Fig. 4a). These data indicate that MKP-5 deficiency reduces mitochondrial ROS production during regenerative myogenesis.

Excessive ROS production in skeletal muscle induces oxidative modification of cellular proteins [45, 46]. We determined the level of oxidative modification of proteins by measurement of cellular lipid peroxidation which occurs as a response to oxidative damage and produces several active aldehydes [45, 47, 48]. The generation of lipid peroxidation in skeletal muscle was determined by measuring the levels of 4-hydroxynonenal (4-HNE)-conjugated proteins. Immunoblotting with anti-4-HNE antibodies showed that the levels of 4-HNE-conjugated proteins were unchanged between uninjured skeletal muscles of *mkp-5* ^*+/+*^ and *mkp-5* ^*−/−*^ mice (Fig. 4b). However, the levels of 4-HNE-conjugated proteins were markedly reduced in regenerating skeletal muscle from *mkp-5* ^*−/−*^ mice as compared with *mkp-5* ^*+/+*^ mice (Fig. 4c). These data demonstrate that MKP-5 deficiency prevents mitochondria-mediated oxidative damage in regenerating skeletal muscle.

To investigate how MKP-5 deficiency attenuates oxidative damage in regenerating skeletal muscle, we assessed the levels of the antioxidant enzyme, catalase, in skeletal muscle from *mkp-5* ^*+/+*^ and *mkp-5* ^*−/−*^ mice. Catalase protects cells from oxidative stress by detoxifying H_2_O_2_ to water [49, 50]. Our results showed that the protein expression levels of catalase were unaltered between *mkp-5* ^*+/+*^ and *mkp-5* ^*−/−*^ mice in uninjured skeletal muscle (Fig. 4d). However, in skeletal muscle of *mkp-5* ^*−/−*^ mice undergoing regeneration, the protein expression levels of catalase were significantly increased as compared with skeletal muscle from *mkp-5* ^*+/+*^ mice at 10 days after injury (Fig. 4e). These findings indicate that mice lacking the expression of MKP-5 are resistant to oxidative cellular damage due to reduced mitochondrial ROS production and commensurate upregulation of catalase during regenerative myogenesis.

### Increased STAT3 activity and Bcl-2 expression in regenerating skeletal muscle of MKP-5-deficient mice

**Fig. 5:**
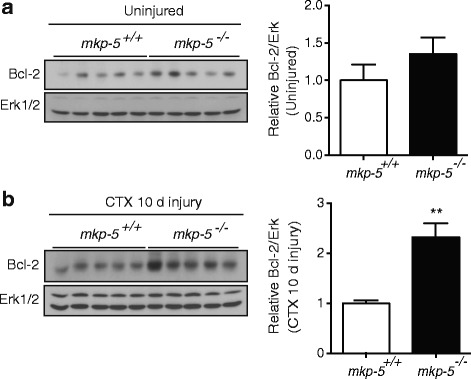
MKP-5-deficient mice exhibit increased Bcl-2 expression in regenerating skeletal muscle. Expression of Bcl-2 protein in *mkp-5* ^*+/+*^ and *mkp-5* ^*−/−*^ mice was determined by immunoblotting with anti-Bcl2 antibodies in muscle lysates prepared from (**a**) uninjured and (**b**) 10 days after CTX-induced injury. The graphs represent quantitation of Bcl-2 immunoblots normalized to Erk1/2 expression. Values are means ± standard errors of the means (SEM). **P < 0.01, *n* = 5 per genotype

To examine the molecular mechanisms of how MKP-5 regulates mitochondria-mediated apoptosis in regenerating skeletal muscle, we assessed the expression of the anti-apoptotic protein, Bcl-2, in skeletal muscle from uninjured and 10 days after injury in *mkp-5* ^*+/+*^ and *mkp-5* ^*−/−*^ mice. As expected, the protein expression of Bcl-2 was unchanged in uninjured skeletal muscle between *mkp-5* ^*+/+*^ and *mkp-5* ^*−/−*^ mice (Fig. 5a). However, at 10 days during regeneration, *mkp-5* ^*−/−*^ mice showed a significant increase in Bcl-2 protein expression as compared with *mkp-5* ^*+/+*^ mice (Fig. 5b). These data indicate that MKP-5 deficiency results in the upregulation of the anti-apoptotic protein Bcl-2 during skeletal muscle regeneration.

**Fig. 6:**
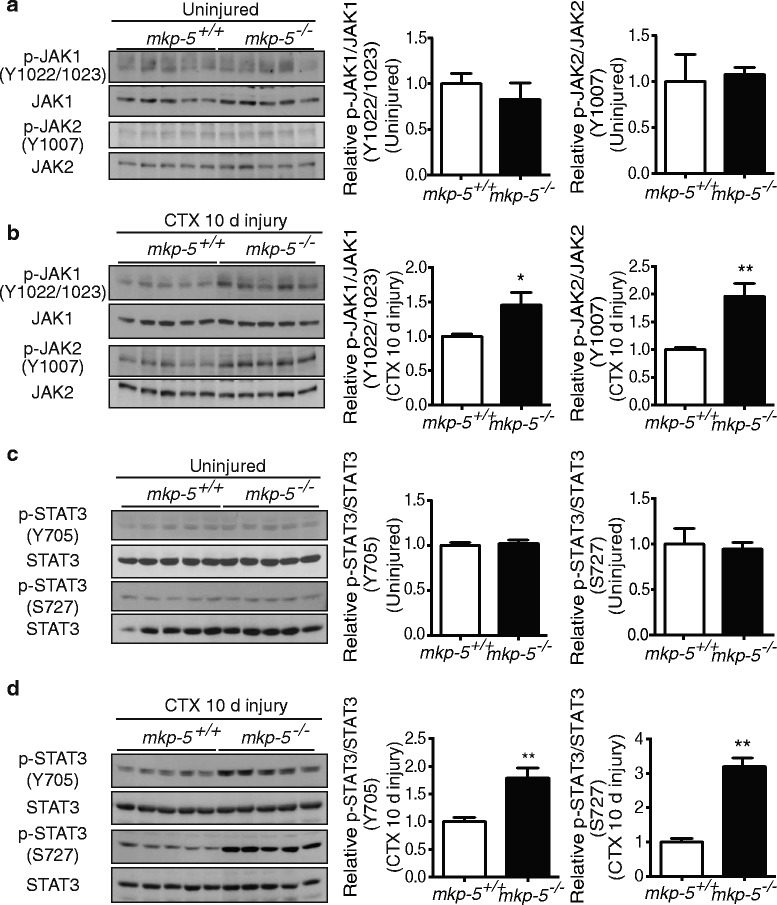
Increased JAK1/2 and STAT3 signaling in regenerating skeletal muscle of MKP-5-deficient mice. Phosphorylation levels of JAK1 (Y1022 /1023) and JAK2 (Y1007) in soleus muscle of (**a**) uninjured and (**b**) 10 days after CTX-induced injury, in *mkp-5* ^*+/+*^ and *mkp-5* ^*−/−*^ mice were determined by immunoblotting with phospho-JAK and total JAK antibodies. Graphs represent the quantitation of immunoblots showing phosphorylated JAK1/2 normalized to JAK1/2 from uninjured and injured skeletal muscle of each genotype. Phosphorylation levels of STAT3 (Y705 and S727) in soleus muscle were determined in (**c**) uninjured and (**d**) 10 days after CTX-induced injury in *mkp-5* ^*+/+*^ and *mkp-5* ^*−/−*^ mice. The graphs represent quantitation of phosphorylated STAT3 normalized to STAT3. Values are means ± standard errors of the means (SEM). *P < 0.05, **P < 0.01, *n* = 5 per genotype

Activation of the JAK/STAT3 pathway has been shown to trigger the expression of anti-apoptotic factors in various tissues [35, 51–53]. Moreover, STAT3 is involved in anti-apoptotic signaling in response to injury [30–32]. Specifically, activated STAT3 promotes Bcl-2 transcription to prevent cells from mitochondria-mediated apoptosis [53–55]. Therefore, we hypothesized that STAT3 may attenuate mitochondria-mediated apoptosis by regulating anti-apoptotic factors in regenerating skeletal muscle of MKP-5-deficient animals. We determined whether MKP-5 regulates phosphorylation of JAK/STAT3 in skeletal muscle. Skeletal muscle from uninjured *mkp-5* ^*+/+*^ and *mkp-5* ^*−/−*^ mice exhibited low, but equivalent, levels of JAK1/2 tyrosyl phosphorylation (Fig. 6a). However, the levels of JAK1/2 tyrosyl phosphorylation were significantly increased in skeletal muscle from *mkp-5* ^*−/−*^ mice at 10 days of regeneration as compared with *mkp-5* ^*+/+*^ mice (Fig. 6b). Consistent with the increased levels of JAK1/2 tyrosyl phosphorylation, the levels of phosphorylation of STAT3 at tyrosine 705 were significantly increased in skeletal muscle from *mkp-5* ^*−/−*^ mice at 10 days of regeneration as compared with *mkp-5* ^*+/+*^ mice (Fig. 6d). Additionally, STAT3 is also phosphorylated at serine 727 and phosphorylation at this site was enhanced at 10 days in regenerating skeletal muscle from *mkp-5* ^*−/−*^ mice as compared with *mkp-5* ^*+/+*^ mice (Fig. 6d). Our results demonstrate that in regenerating skeletal muscle, MKP-5 deficiency triggers the upregulation of JAK/STAT3 signaling. Furthermore, these results are consistent with the observation that the STAT3 target, Bcl-2, is concomitantly increased in regenerating skeletal muscle of MKP-5-deficient mice.

### MKP-5 mediates STAT3/Bcl-2 in a JNK and p38 MAPK-dependent manner

**Fig. 7:**
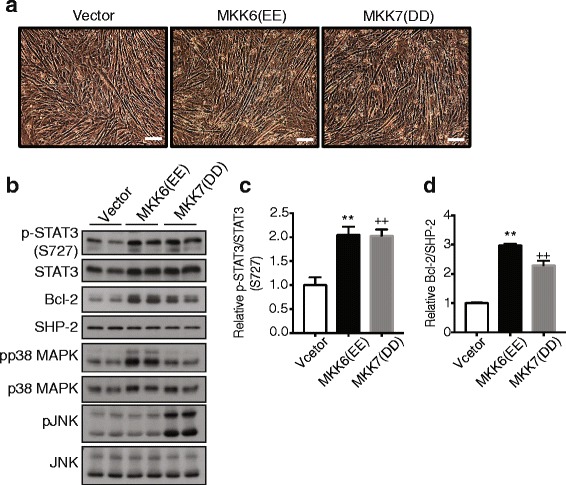
Activation of p38 MAPK and JNK induce the phosphorylation of STAT3 (S727) and Bcl-2 expression. C2C12 myoblasts were transfected with either vector control, constitutively active mutants of MKK6(EE) or MKK7(DD). After 24 h, myoblasts were induced to undergo differentiation for 48 h. (**a**) Representative images of differentiated C2C12 myoblasts for 48 h. (**b**) Transfected differentiated C2C12 myotubes were lysed and immunoblotting for the expression of the indicated proteins. Graph showing quantitation of (**c**) phosphorylation levels of STAT3 (S727) and (**d**) Bcl-2 protein expression. The graphs represent quantitation of phosphorylated STAT3 normalized to STAT3, and quantitation of Bcl-2 proteins normalized to SHP-2. Values are means ± standard errors of the means (SEM) and were analyzed by ANOVA with Tukey’s multiple comparison test. Two asterisks indicate MKK6(EE) significantly different vs. vector (*P* < 0.01). Two plus signs indicate MKK7(DD) significantly different vs vector (*P* < 0.01)

Our data demonstrate that MKP-5 mediates STAT3 phosphorylation at both tyrosine 705 and serine 727 residues (Fig. 6). STAT3 is tyrosine phosphorylated by JAK1/2, and its levels are increased in the absence of MKP-5 suggesting that MKP-5 indirectly regulates STAT3 tyrosyl phosphorylation through JAK1/2. Additionally, STAT3 is increased in its levels of serine 727 phosphorylation (Fig. 6). It is known that MAPKs phosphorylate STAT3 at this serine site resulting in optimal STAT3 transcriptional activity [37, 56]. In order to identity whether MKP-5 regulation of either p38 MAPK and/or JNK is responsible for the phosphorylation of STAT3 at serine 727, we used a constitutively active mutant of MKK6 and MKK7 to induce p38 MAPK and JNK activity, respectively, in differentiated C2C12 myoblasts. We found that both activated mutants of MKK6 and MKK7 resulted in increased levels of STAT3 serine 727 phosphorylation and increased protein expression of Bcl-2 (Fig. 7a–d) in terminally differentiated C2C12 myotubes. These results indicate that both p38 MAPK and JNK are capable of inducing STAT3 serine 727 phosphorylation and upregulation of Bcl-2 protein expression in differentiated myotubes.

## Discussion

**Fig. 8:**
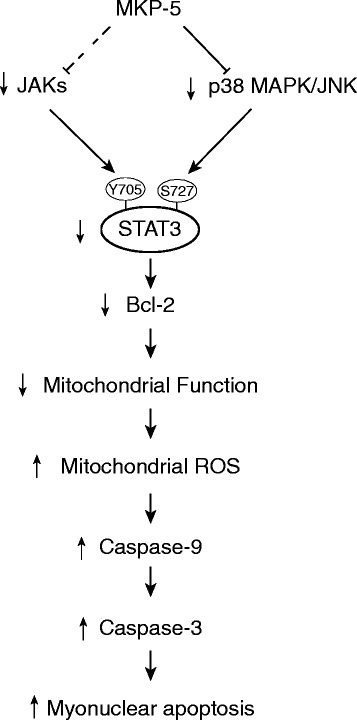
Model for the mechanism of MKP-5 in myonuclear apoptosis. MKP-5 negatively regulates mitochondria-mediated myonuclear apoptosis in response to injury. See text for additional details

MKP-5 is a dual-specificity protein tyrosine phosphatase that specifically dephosphorylates and inactivates p38 MAPK and JNK1/2 but not ERK1/2 [4, 57]. It has been established that p38 MAPK and JNK play essential roles in skeletal muscle myogenesis and regeneration [4–7]. We have demonstrated in skeletal muscle that MKP-5 negatively regulates both p38 MAPK and JNK, but not ERK1/2 [4, 13, 14]. MKP-5 is highly expressed in skeletal muscle and is differentially regulated in response to specific stimuli such as cellular injury [4, 13, 14]. Our group has demonstrated that MKP-5 is an essential negative regulator of SC proliferation and differentiation [4, 58]. SCs endow skeletal muscle with its highly regenerative capacity, and the process of regeneration in response to injury is orchestrated by synchronizing the activation of various cellular responses [59, 60]. The MAPKs have also been shown to be involved in regulating myofiber survival [61, 62]. In skeletal muscle, p38 MAPK and JNK are negatively regulated by MKP-5, which contributes to the maintenance of myofiber homeostasis [4, 58]. During muscle regeneration, MKP-5 is downregulated, thereby promoting p38 MAPK/JNK signaling and subsequent repair of damaged muscle [4]. However, the effect of MKP-5 on myofiber survival during regeneration is unclear. Here, in this report, we show that MKP-5 deficiency promotes myofiber survival during regeneration through a pathway involving JAK/STAT3 and mitochondria-mediated apoptotic signaling (Fig. 8).

During skeletal muscle repair, MKP-5 becomes downregulated in the myofiber in order to coordinate the repair of skeletal muscle [4]. As such, mice lacking MKP-5 exhibit enhanced regenerative myogenesis which is also accompanied by increased SC proliferation and differentiation [4]. Here, we show that the enhanced regenerative capacity of MKP-5-deficient mice is also a result of improved myofiber survival (Fig. 8). Consistent with the notion that transient MKP-5 downregulation plays an important role in muscle repair [4], we found that MKP-5-deficient myofibers show decreased levels of apoptosis. Myonuclear apoptosis is coordinated by the activation of caspases that play essential roles in cell survival and death. Caspases can be activated through the mitochondria-mediated (intrinsic) and/or the death receptor-mediated (extrinsic) pathways [27, 63]. Specifically, caspase-3 is a protease capable of degrading intact actomyosin proteins, promoting DNA fragmentation and myonuclear apoptosis [18]. Our findings showed that activation of caspase-3 was attenuated in MKP-5-deficient skeletal muscle during regeneration. In addition to attenuated activation of caspase-3, MKP-5-deficient skeletal muscle displayed decreased activation of caspase-9 triggered by the intrinsic, but not caspase-8, induced by the extrinsic pathway. These findings support the notion that during regenerative myogenesis MKP-5 downregulation is necessary to engage the intrinsic, caspase-3-dependent pathway, in order to maintain myofiber survival.

It is well established that mitochondria play a central role in both regeneration and skeletal muscle apoptosis [18–20]. Moreover, mitochondrial dysfunction underlies numerous diseases including those of skeletal muscle [18, 19, 40]. In this study, we have identified a novel link between MKP-5 and mitochondrial function. MKP-5-deficient mice showed significantly enhanced mitochondrial biogenesis and mitochondrial respiratory function in regenerating skeletal muscle. Furthermore, it has been demonstrated that in skeletal muscle, mitochondrial dysfunction is associated with excessive ROS production [18, 19, 40]. Excessive mitochondrial ROS production leads to the release of cytochrome c in to the cytoplasm through permeabilization of the outer mitochondrial membrane [64–66]. Released cytochrome c forms the apoptosome complex with the apoptotic protease-activating factor 1, which promotes activation of caspase-9 resulting in cleavage of caspase-3 [67, 68]. Given that MKP-5 deficiency promotes mitochondrial respiratory function in skeletal muscle, we tested the hypothesis that the loss of MKP-5 attenuates increased mitochondrial ROS production during skeletal muscle regeneration. Our data demonstrate that MKP-5-deficient mice significantly attenuated mitochondria-derived hydrogen peroxide production in regenerative skeletal muscle. Excessive ROS production induced by dysfunctional mitochondria results in oxidative tissue damage [18, 40]. Specifically, lipid peroxidation occurs as a response to oxidative stress and produces several biologically active aldehydes that result in oxidative injury to tissues [45, 48]. It has been shown that 4-HNE-conjugated proteins are the most abundant unsaturated aldehydes and accumulate in diseased and injured tissues [48]. The results of the present study showed that MKP-5-deficient skeletal muscle is resistant to the accumulation of 4-HNE-conjugated protein adduct during skeletal muscle regeneration. Concomitant with the resistance to ROS-mediated damage, we found that MKP-5-deficient skeletal muscle expressed increased catalase levels. Interestingly, it has been reported that neutrophils and macrophages derived from *mkp-5* ^*−/−*^ mice produce more superoxide as compared to wild type animals in response to certain stimuli [69, 70]. However, here, we observed reduced production of hydrogen peroxide from skeletal muscle-derived mitochondria and subsequent oxidation-driven protein damage in skeletal muscle of *mkp-5* ^*−/−*^ mice. The nature for how MKP-5 appears to have differential effects of ROS generation in hematopoietic-derived cells as compared with skeletal muscle is yet to be understood fully. Nevertheless, these findings indicate that in skeletal muscle, MKP-5-deficient mice are protected from oxidative tissue damage as a result of both improved mitochondrial function and efficiency of antioxidant capacity in regenerative skeletal muscle. Given that MKP-5 becomes downregulated early on during regenerative myogenesis, these results further suggest that MKP-5 in skeletal muscle plays an important role in mediating critical survival pathways that are required for the successful regeneration of skeletal muscle.

STAT3 becomes activated in response to both tyrosine and serine phosphorylation [30, 53, 54]. Once activated, STAT3 stimulates the transcription of a number of targets including anti-apoptotic protein Bcl-2. STAT3-induced transcriptional activation of Bcl-2 has been shown to protect mitochondria from a variety of cellular stresses [51, 53, 54, 71]. We found that in regenerating skeletal muscle of MKP-5-deficient mice that both JAK1 and JAK2 were enhanced in their levels of activity and subsequently, STAT3 tyrosine 705 phosphorylation was concomitantly upregulated. These results imply that MKP-5 negatively regulates STAT3 activity. STAT3 is also phosphorylated on serine 727 which has been shown to be required for optimal STAT3 transcriptional activation [56, 72]. Given that MKP-5 negatively regulates p38 MAPK/JNK, we examined whether STAT3 serine 727 phosphorylation was also upregulated in the absence of MKP-5 in skeletal muscle. Interestingly, serine 727 was indeed upregulated on STAT3. These findings further support the supposition that MKP-5 negatively regulates STAT3 function and thus, downstream activation of STAT3 target genes, such as Bcl2. The consequences of increased Bcl-2 expression in skeletal muscle of MKP-5-deficient mice are consistent with the observation that these mice exhibit improved levels of survival during regenerative myogenesis [4]. Overall, multiple lines of evidence support the idea that MKP-5 plays an important role in maintaining skeletal muscle homeostasis as shown here by regulating skeletal muscle health.

Recently, we have demonstrated that MKP-5 participates in a signaling pathway that mediates the secretion of IL-6 and mice lacking MKP-5 express increased circulating IL-6 levels. Our results confirm this previous report that MKP-5-deficient mice exhibit increased STAT3 tyrosine phosphorylation but also extend that to show serine 727 phosphorylation on STAT3 is also increased. It is reasonable to propose that the increased circulating levels of IL-6 in MKP-5-deficient mice are, at least in part, responsible for the enhanced levels of JAK1/2 activation. However, it is formally possible that MKP-5-deficiency may act in a parallel pathway to influence the activation of JAK1/2 in response to other growth factor and/or cytokines. Further experiments will be needed to clarify the precise mechanism through which MKP-5 regulates JAK/STAT3 signaling in skeletal muscle.

## Conclusion

This study provides important insight into the mechanisms of MKP-5 function in skeletal muscle homeostasis and regeneration. Our results demonstrate that MKP-5 coordinates the activation of p38 MAPK/JNK signaling that couples to Bcl-2-mediated regulation of apoptosis. Furthermore, MKP-5 deficiency protects against skeletal muscle death through pathways that involve improved mitochondria-mediated handling of oxidative stress. Together, these data provide further evidence that MKP-5 antagonism may represent a novel therapeutic approach for the preservation and/or maintenance of skeletal muscle health in dystrophic skeletal muscle diseases.

## Abbreviations

4-HNE: 4-Hydroxynonenal
CTX: Cardiotoxin
DUSP: Dual-specificity phosphatase
MAPK: Mitogen-activated protein kinase
MKP-5: Mitogen-activated protein kinase phosphatase-5
ROS: Reactive oxygen species
SCs: Satellite cells
STAT3: Signal transducer and activator of transcription 3
TUNEL: Terminal deoxynucleotidyl transferase dUTP nick end labeling

## References

[1] Santos SD, Verveer PJ, Bastiaens PI (2007). Growth factor-induced MAPK network topology shapes Erk response determining PC-12 cell fate. Nat Cell Biol.

[2] Puigserver P, Rhee J, Lin J, Wu Z, Yoon JC, Zhang CY, Krauss S, Mootha VK, Lowell BB, Spiegelman BM (2001). Cytokine stimulation of energy expenditure through p38 MAP kinase activation of PPARgamma coactivator-1. Mol Cell.

[3] Seko Y, Takahashi N, Tobe K, Kadowaki T, Yazaki Y (1997). Hypoxia and hypoxia/reoxygenation activate p65PAK, p38 mitogen-activated protein kinase (MAPK), and stress-activated protein kinase (SAPK) in cultured rat cardiac myocytes. Biochem Biophys Res Commun.

[4] Shi H, Verma M, Zhang L, Dong C, Flavell RA, Bennett AM (2013). Improved regenerative myogenesis and muscular dystrophy in mice lacking Mkp5. J Clin Invest.

[5] Cuenda A, Cohen P (1999). Stress-activated protein kinase-2/p38 and a rapamycin-sensitive pathway are required for C2C12 myogenesis. J Biol Chem.

[6] Perdiguero E, Ruiz-Bonilla V, Gresh L, Hui L, Ballestar E, Sousa-Victor P, Baeza-Raja B, Jardi M, Bosch-Comas A, Esteller M (2007). Genetic analysis of p38 MAP kinases in myogenesis: fundamental role of p38alpha in abrogating myoblast proliferation. EMBO J.

[7] Cheung TH, Rando TA (2013). Molecular regulation of stem cell quiescence. Nat Rev Mol Cell Biol.

[8] Goodyear LJ, Chang PY, Sherwood DJ, Dufresne SD, Moller DE (1996). Effects of exercise and insulin on mitogen-activated protein kinase signaling pathways in rat skeletal muscle. Am J Phys.

[9] Lawan A, Shi H, Gatzke F, Bennett AM (2013). Diversity and specificity of the mitogen-activated protein kinase phosphatase-1 functions. Cell Mol Life Sci.

[10] Soulsby M, Bennett AM (2009). Physiological signaling specificity by protein tyrosine phosphatases. Physiology (Bethesda).

[11] Dickinson RJ, Keyse SM (2006). Diverse physiological functions for dual-specificity MAP kinase phosphatases. J Cell Sci.

[12] Boutros T, Chevet E, Metrakos P (2008). Mitogen-activated protein (MAP) kinase/MAP kinase phosphatase regulation: roles in cell growth, death, and cancer. Pharmacol Rev.

[13] Theodosiou A, Smith A, Gillieron C, Arkinstall S, Ashworth A (1999). MKP5, a new member of the MAP kinase phosphatase family, which selectively dephosphorylates stress-activated kinases. Oncogene.

[14] Masuda K, Shima H, Kikuchi K, Watanabe Y, Matsuda Y (2000). Expression and comparative chromosomal mapping of MKP-5 genes DUSP10/Dusp10. Cytogenet Cell Genet.

[15] Tamilarasan KP, Temmel H, Das SK, Al Zoughbi W, Schauer S, Vesely PW, Hoefler G (2012). Skeletal muscle damage and impaired regeneration due to LPL-mediated lipotoxicity. Cell Death Dis.

[16] Jejurikar SS, Kuzon WM (2003). Satellite cell depletion in degenerative skeletal muscle. Apoptosis.

[17] Demontis F, Piccirillo R, Goldberg AL, Perrimon N (2013). Mechanisms of skeletal muscle aging: insights from Drosophila and mammalian models. Dis Model Mech.

[18] Min K, Kwon OS, Smuder AJ, Wiggs MP, Sollanek KJ, Christou DD, Yoo JK, Hwang MH, Szeto HH, Kavazis AN, Powers SK (2015). Increased mitochondrial emission of reactive oxygen species and calpain activation are required for doxorubicin-induced cardiac and skeletal muscle myopathy. J Physiol.

[19] Min K, Smuder AJ, Kwon OS, Kavazis AN, Szeto HH, Powers SK (1985). Mitochondrial-targeted antioxidants protect skeletal muscle against immobilization-induced muscle atrophy. J Appl Physiol.

[20] Rodgers JT, King KY, Brett JO, Cromie MJ, Charville GW, Maguire KK, Brunson C, Mastey N, Liu L, Tsai CR (2014). mTORC1 controls the adaptive transition of quiescent stem cells from G0 to G(Alert). Nature.

[21] LaBarge S, McDonald M, Smith-Powell L, Auwerx J, Huss JM (2014). Estrogen-related receptor-alpha (ERRalpha) deficiency in skeletal muscle impairs regeneration in response to injury. FASEB J.

[22] Wagatsuma A, Kotake N, Yamada S (2011). Muscle regeneration occurs to coincide with mitochondrial biogenesis. Mol Cell Biochem.

[23] Duguez S, Feasson L, Denis C, Freyssenet D (2002). Mitochondrial biogenesis during skeletal muscle regeneration. Am J Physiol Endocrinol Metab.

[24] Rybalka E, Timpani CA, Cooke MB, Williams AD, Hayes A (2014). Defects in mitochondrial ATP synthesis in dystrophin-deficient mdx skeletal muscles may be caused by complex I insufficiency. PLoS One.

[25] Passaquin AC, Renard M, Kay L, Challet C, Mokhtarian A, Wallimann T, Ruegg UT (2002). Creatine supplementation reduces skeletal muscle degeneration and enhances mitochondrial function in mdx mice. Neuromuscul Disord.

[26] Lin MT, Beal MF (2006). Mitochondrial dysfunction and oxidative stress in neurodegenerative diseases. Nature.

[27] Elmore S (2007). Apoptosis: a review of programmed cell death. Toxicol Pathol.

[28] Haunstetter A, Izumo S (1998). Apoptosis: basic mechanisms and implications for cardiovascular disease. Circ Res.

[29] Ghavami S, Shojaei S, Yeganeh B, Ande SR, Jangamreddy JR, Mehrpour M, Christoffersson J, Chaabane W, Moghadam AR, Kashani HH (2014). Autophagy and apoptosis dysfunction in neurodegenerative disorders. Prog Neurobiol.

[30] Zhang C, Li Y, Wu Y, Wang L, Wang X, Du J (2013). Interleukin-6/signal transducer and activator of transcription 3 (STAT3) pathway is essential for macrophage infiltration and myoblast proliferation during muscle regeneration. J Biol Chem.

[31] Golding JP, Calderbank E, Partridge TA, Beauchamp JR (2007). Skeletal muscle stem cells express anti-apoptotic ErbB receptors during activation from quiescence. Exp Cell Res.

[32] Hilfiker-Kleiner D, Hilfiker A, Fuchs M, Kaminski K, Schaefer A, Schieffer B, Hillmer A, Schmiedl A, Ding Z, Podewski E (2004). Signal transducer and activator of transcription 3 is required for myocardial capillary growth, control of interstitial matrix deposition, and heart protection from ischemic injury. Circ Res.

[33] Lee H, Min K, Yi JS, Shi H, Chang W, Jackson L, Bennett AM (2017). A phosphoproteomic screen identifies a guanine nucleotide exchange factor for Rab3A protein as a mitogen-activated protein (MAP) kinase phosphatase-5-regulated MAP kinase target in interleukin 6 (IL-6) secretion and myogenesis. J Biol Chem.

[34] Kumar J, Ward AC (1845). Role of the interleukin 6 receptor family in epithelial ovarian cancer and its clinical implications. Biochim Biophys Acta.

[35] Haga S, Terui K, Zhang HQ, Enosawa S, Ogawa W, Inoue H, Okuyama T, Takeda K, Akira S, Ogino T (2003). Stat3 protects against Fas-induced liver injury by redox-dependent and -independent mechanisms. J Clin Invest.

[36] Hardwick JM, Soane L. Multiple functions of BCL-2 family proteins. Cold Spring Harb Perspect Biol. 2013;5(2):a008722. 10.1101/cshperspect.a008722 PMC3552500 23378584

[37] Benekli M, Baumann H, Wetzler M (2009). Targeting signal transducer and activator of transcription signaling pathway in leukemias. J Clin Oncol.

[38] Zhang Y, Blattman JN, Kennedy NJ, Duong J, Nguyen T, Wang Y, Davis RJ, Greenberg PD, Flavell RA, Dong C (2004). Regulation of innate and adaptive immune responses by MAP kinase phosphatase 5. Nature.

[39] Yamashita N, Hoshida S, Otsu K, Asahi M, Kuzuya T, Hori M (1999). Exercise provides direct biphasic cardioprotection via manganese superoxide dismutase activation. J Exp Med.

[40] Powers SK, Hudson MB, Nelson WB, Talbert EE, Min K, Szeto HH, Kavazis AN, Smuder AJ (2011). Mitochondria-targeted antioxidants protect against mechanical ventilation-induced diaphragm weakness. Crit Care Med.

[41] Wang C, Youle RJ (2009). The role of mitochondria in apoptosis*. Annu Rev Genet.

[42] Lee Y, Min K, Talbert EE, Kavazis AN, Smuder AJ, Willis WT, Powers SK (2012). Exercise protects cardiac mitochondria against ischemia-reperfusion injury. Med Sci Sports Exerc.

[43] Fleury C, Mignotte B, Vayssiere JL (2002). Mitochondrial reactive oxygen species in cell death signaling. Biochimie.

[44] Adhihetty PJ, O'Leary MF, Chabi B, Wicks KL, Hood DA (2007). Effect of denervation on mitochondrially mediated apoptosis in skeletal muscle. J Appl Physiol (1985).

[45] Smuder AJ, Kavazis AN, Min K, Powers SK (2011). Exercise protects against doxorubicin-induced oxidative stress and proteolysis in skeletal muscle. J Appl Physiol (1985).

[46] Pedersen WA, Fu W, Keller JN, Markesbery WR, Appel S, Smith RG, Kasarskis E, Mattson MP (1998). Protein modification by the lipid peroxidation product 4-hydroxynonenal in the spinal cords of amyotrophic lateral sclerosis patients. Ann Neurol.

[47] Pizzimenti S, Ciamporcero E, Daga M, Pettazzoni P, Arcaro A, Cetrangolo G, Minelli R, Dianzani C, Lepore A, Gentile F, Barrera G (2013). Interaction of aldehydes derived from lipid peroxidation and membrane proteins. Front Physiol.

[48] Vladykovskaya E, Sithu SD, Haberzettl P, Wickramasinghe NS, Merchant ML, Hill BG, McCracken J, Agarwal A, Dougherty S, Gordon SA (2012). Lipid peroxidation product 4-hydroxy-trans-2-nonenal causes endothelial activation by inducing endoplasmic reticulum stress. J Biol Chem.

[49] DeJong RJ, Miller LM, Molina-Cruz A, Gupta L, Kumar S, Barillas-Mury C (2007). Reactive oxygen species detoxification by catalase is a major determinant of fecundity in the mosquito Anopheles gambiae. Proc Natl Acad Sci U S A.

[50] Barbosa MR, Sampaio IH, Teodoro BG, Sousa TA, Zoppi CC, Queiroz AL, Passos MA, Alberici LC, Teixeira FR, Manfiolli AO (2013). Hydrogen peroxide production regulates the mitochondrial function in insulin resistant muscle cells: effect of catalase overexpression. Biochim Biophys Acta.

[51] Jacoby JJ, Kalinowski A, Liu MG, Zhang SS, Gao Q, Chai GX, Ji L, Iwamoto Y, Li E, Schneider M (2003). Cardiomyocyte-restricted knockout of STAT3 results in higher sensitivity to inflammation, cardiac fibrosis, and heart failure with advanced age. Proc Natl Acad Sci U S A.

[52] Negoro S, Kunisada K, Tone E, Funamoto M, Oh H, Kishimoto T, Yamauchi-Takihara K (2000). Activation of JAK/STAT pathway transduces cytoprotective signal in rat acute myocardial infarction. Cardiovasc Res.

[53] Bhattacharya S, Ray RM, Johnson LR (2005). STAT3-mediated transcription of Bcl-2, Mcl-1 and c-IAP2 prevents apoptosis in polyamine-depleted cells. Biochem J.

[54] Choi HJ, Lee JH, Park SY, Cho JH, Han JS (2009). STAT3 is involved in phosphatidic acid-induced Bcl-2 expression in HeLa cells. Exp Mol Med.

[55] Hattori R, Maulik N, Otani H, Zhu L, Cordis G, Engelman RM, Siddiqui MA, Das DK (2001). Role of STAT3 in ischemic preconditioning. J Mol Cell Cardiol.

[56] Wen Z, Zhong Z, Darnell JE (1995). Maximal activation of transcription by Stat1 and Stat3 requires both tyrosine and serine phosphorylation. Cell.

[57] Tanoue T, Moriguchi T, Nishida E (1999). Molecular cloning and characterization of a novel dual specificity phosphatase, MKP-5. J Biol Chem.

[58] Shi H, Gatzke F, Molle JM, Lee HB, Helm ET, Oldham JJ, Zhang L, Gerrard DE, Bennett AM (2015). Mice lacking MKP-1 and MKP-5 reveal hierarchical regulation of regenerative myogenesis. J Stem Cell Regen Biol.

[59] Charge SB, Rudnicki MA (2004). Cellular and molecular regulation of muscle regeneration. Physiol Rev.

[60] Shi X, Garry DJ (2006). Muscle stem cells in development, regeneration, and disease. Genes Dev.

[61] Ronda AC, Vasconsuelo A, Boland R (2010). Extracellular-regulated kinase and p38 mitogen-activated protein kinases are involved in the antiapoptotic action of 17beta-estradiol in skeletal muscle cells. J Endocrinol.

[62] Ostrovsky O, Bengal E (2003). The mitogen-activated protein kinase cascade promotes myoblast cell survival by stabilizing the cyclin-dependent kinase inhibitor, p21WAF1 protein. J Biol Chem.

[63] Primeau AJ, Adhihetty PJ, Hood DA (2002). Apoptosis in heart and skeletal muscle. Can J Appl Physiol.

[64] Suzuki S, Higuchi M, Proske RJ, Oridate N, Hong WK, Lotan R (1999). Implication of mitochondria-derived reactive oxygen species, cytochrome C and caspase-3 in N-(4-hydroxyphenyl)retinamide-induced apoptosis in cervical carcinoma cells. Oncogene.

[65] Petrosillo G, Ruggiero FM, Pistolese M, Paradies G (2001). Reactive oxygen species generated from the mitochondrial electron transport chain induce cytochrome c dissociation from beef-heart submitochondrial particles via cardiolipin peroxidation. Possible role in the apoptosis. FEBS Lett.

[66] Petrosillo G, Ruggiero FM, Paradies G (2003). Role of reactive oxygen species and cardiolipin in the release of cytochrome c from mitochondria. FASEB J.

[67] Circu ML, Aw TY (2010). Reactive oxygen species, cellular redox systems, and apoptosis. Free Radic Biol Med.

[68] Li P, Nijhawan D, Budihardjo I, Srinivasula SM, Ahmad M, Alnemri ES, Wang X (1997). Cytochrome c and dATP-dependent formation of Apaf-1/caspase-9 complex initiates an apoptotic protease cascade. Cell.

[69] Qian F, Deng J, Gantner BN, Flavell RA, Dong C, Christman JW, Ye RD (2012). Map kinase phosphatase 5 protects against sepsis-induced acute lung injury. Am J Physiol Lung Cell Mol Physiol.

[70] Qian F, Deng J, Cheng N, Welch EJ, Zhang Y, Malik AB, Flavell RA, Dong C, Ye RD (2009). A non-redundant role for MKP5 in limiting ROS production and preventing LPS-induced vascular injury. EMBO J.

[71] Shen Y, Devgan G, Darnell JE, Bromberg JF (2001). Constitutively activated Stat3 protects fibroblasts from serum withdrawal and UV-induced apoptosis and antagonizes the proapoptotic effects of activated Stat1. Proc Natl Acad Sci U S A.

[72] Decker T, Kovarik P (2000). Serine phosphorylation of STATs. Oncogene.

